# Boosting fast energy storage by synergistic engineering of carbon and deficiency

**DOI:** 10.1038/s41467-019-13945-1

**Published:** 2020-01-09

**Authors:** Shengjue Deng, He Zhu, Guizhen Wang, Mi Luo, Shenghui Shen, Changzhi Ai, Liang Yang, Shiwei Lin, Qinghua Zhang, Lin Gu, Bo Liu, Yan Zhang, Qi Liu, Guoxiang Pan, Qinqin Xiong, Xiuli Wang, Xinhui Xia, Jiangping Tu

**Affiliations:** 1 State Key Laboratory of Silicon Materials, Key Laboratory of Advanced Materials and Applications for Batteries of Zhejiang Province, and School of Materials Science & Engineering, Zhejiang University, Hangzhou, 310027 PR China; 2 Department of Physics, City University of Hong Kong, Hong Kong, 999077 PR China; 3 State Key Laboratory of Marine Resource Utilization in South China Sea, Hainan University, Haikou, 570228 PR China; 4 Shanghai Synchrotron Radiation Facility, Shanghai Institute of Applied Physics, Chinese Academy of Sciences, Shanghai, 201210 PR China; 5 Institute of Physics, Chinese Academy of Sciences, Beijing, 100190 PR China; 6 Shenzhen Research Institute, City University of Hong Kong, Shenzhen, 518057 PR China; 7 Department of Materials Chemistry, Huzhou University, Huzhou, 313000 PR China; 8 College of Materials and Environmental Engineering, Hangzhou Dianzi University, Hangzhou, 310018 Zhejiang PR China

**Keywords:** Nanoscience and technology, Nanoparticles

## Abstract

Exploring advanced battery materials with fast charging/discharging capability is of great significance to the development of modern electric transportation. Herein we report a powerful synergistic engineering of carbon and deficiency to construct high-quality three/two-dimensional cross-linked Ti_2_Nb_10_O_29−*x*_@C composites at primary grain level with conformal and thickness-adjustable boundary carbon. Such exquisite boundary architecture is demonstrated to be capable of regulating the mechanical stress and concentration of oxygen deficiency for desired performance. Consequently, significantly improved electronic conductivity and enlarged lithium ion diffusion path, shortened activation process and better structural stability are realized in the designed Ti_2_Nb_10_O_29−*x*_@C composites. The optimized Ti_2_Nb_10_O_29−*x*_@C composite electrode shows fast charging/discharging capability with a high capacity of 197 mA h g^−1^ at 20 C (∼3 min) and excellent long-term durability with 98.7% electron and Li capacity retention over 500 cycles. Most importantly, the greatest applicability of our approach has been demonstrated by various other metal oxides, with tunable morphology, structure and composition.

## Abstract (web summary)

High-rate electrode materials are the key to fast-charging batteries that can store large quantities of charge in minutes or even seconds. Here the authors introduce adaptive carbon layer to oxygen defective Ti_2_Nb_10_O_29_ forming composite electrodes that deliver impressive rate performance and stability.

## Introduction

In today’s society, lithium ion batteries (LIBs) are continuing to boom to meet the growing demand of smart electronics (i.e., smartphones, laptops, etc.) and electric vehicles (EVs)^1,2^. With sales rising of EVs and demand for smartphones slowing, it is reasonable to believe that EVs will dominate the demand for LIBs in the near future. For a decade, with the adoption of high-energy electrode materials, LIB technology is developing rapidly and new EV models are being rolled out with much improved mileage range. However, compared with mileage range, charging time in a few minutes, rather than hours, has already become the major concern in the EV industry. Apparently, exploring high-power electrode materials with fast charge/discharge capability is highly desirable to alleviate this technological challenge and enable the development of new power-intensive devices^3–6^.

The high-speed storage of electrical energy critically depends on the facile transport of Li ions and electrons in the electrode materials, for which the improvement of the lithium mobility and electronic conductivity is the key of success. Currently, various techniques and approaches have been developed in order to achieve the high-rate electrodes. Among them, the strategy of building nanostructured materials is the most direct and effective one, because the increased surface area can provide more active sites in contact with electrolyte, and the reduced length of diffusion path also fastens the transport of electron and Li^+^^7–9^. However, this strategy alone still makes it difficult for the electrode materials to perform well because most battery electrodes have intrinsically low electrical conductivity. In such a context, another important approach, carbon coating, has also been frequently used in both bulk and nanostructured electrodes to optimize the power capability. To date, numerous carbon-coated electrodes, such as LiFePO_4_/C^10^ and Si/C^11^, have been developed via various technologies including atomic layer deposition (ALD)^12^, molecular layer deposition (MLD)^13^, hydrothermal^14^, electrodeposition^15^, and chemical vapor deposition^16,17^. Despite the substantial efforts, however, these carbon-coating techniques are still far away from the requirements of industry for their drawbacks, such as high cost and complex synthetic control. For example, ALD and MLD methods fabricate carbon shell from converting organic polyamide film, which usually involves high-temperature treatment (>600 ^o^C) and long-time operation. In addition, such uneconomical process has also been frequently used in other carbon-coating techniques like hydrothermal and electrochemical depositions. Furthermore, previous studies have been mainly focused on the uniform carbon coating at the secondary-particle level, which can only take effect on the enhancement of intrinsic conductivity and alleviation of mechanical stress with varying degree of success. Considering the power capability of LIBs depends extremely on the enhanced intrinsic conductivity of every individual grain, carbon coating at the primary-grain level will inherently result in the reinforced rate capability. Given all that, it is highly desirable to fully take advantages of both nanostructuring and carbon-coating techniques. And meanwhile to develop a facile and practical strategy, which can realize boundary carbon coating, which means that the carbon can coat on the active materials at primary-grain level with good conformity and adjustable thickness.

In this work, a facile and controllable low-temperature carbon-coating technique has been developed by controlling acetylene decomposition process. In a departure from other approaches, our strategy is capable of creating a uniform carbon coating with desired thickness at primary-grain level, which inherently results in high charge and discharge rate in the nanostructured electrode. As an instance, we demonstrate that with this appropriate low-temperature treatment, the conductive carbon with tunable thickness can be uniformly coated on boundary of nanostructured Ti_2_Nb_10_O_29_ (TNO), allowing the rationally designed Ti_2_Nb_10_O_29−*x*_@C (TNO_−*x*_@C) composites to be used at extremely high rates (197 mA h g^−1^ at 20 C, corresponding to a charge time of 3 min). Compared with the pure TNO, the significantly improved performance can be ascribed to the enhanced electronic conductivity, improved Li ion diffusion properties and unexpected defective chemistry. Also, our in situ high-energy synchrotron X-ray diffraction (HEXRD) results show that the lattice anisotropy of TNO_−*x*_@C has also been significantly weakened during the operation. Importantly, we further demonstrate the generality of this uniform engineering of carbon, which can be applied to achieve other high-rate metal oxides such as Nb_2_O_5−*x*_@C microrods, TiO_2−*x*_@C nanoparticles and ZnO_−*x*_@C nanorods.

## Results

### Synthesis and structural characterization of the products

**Fig. 1:**
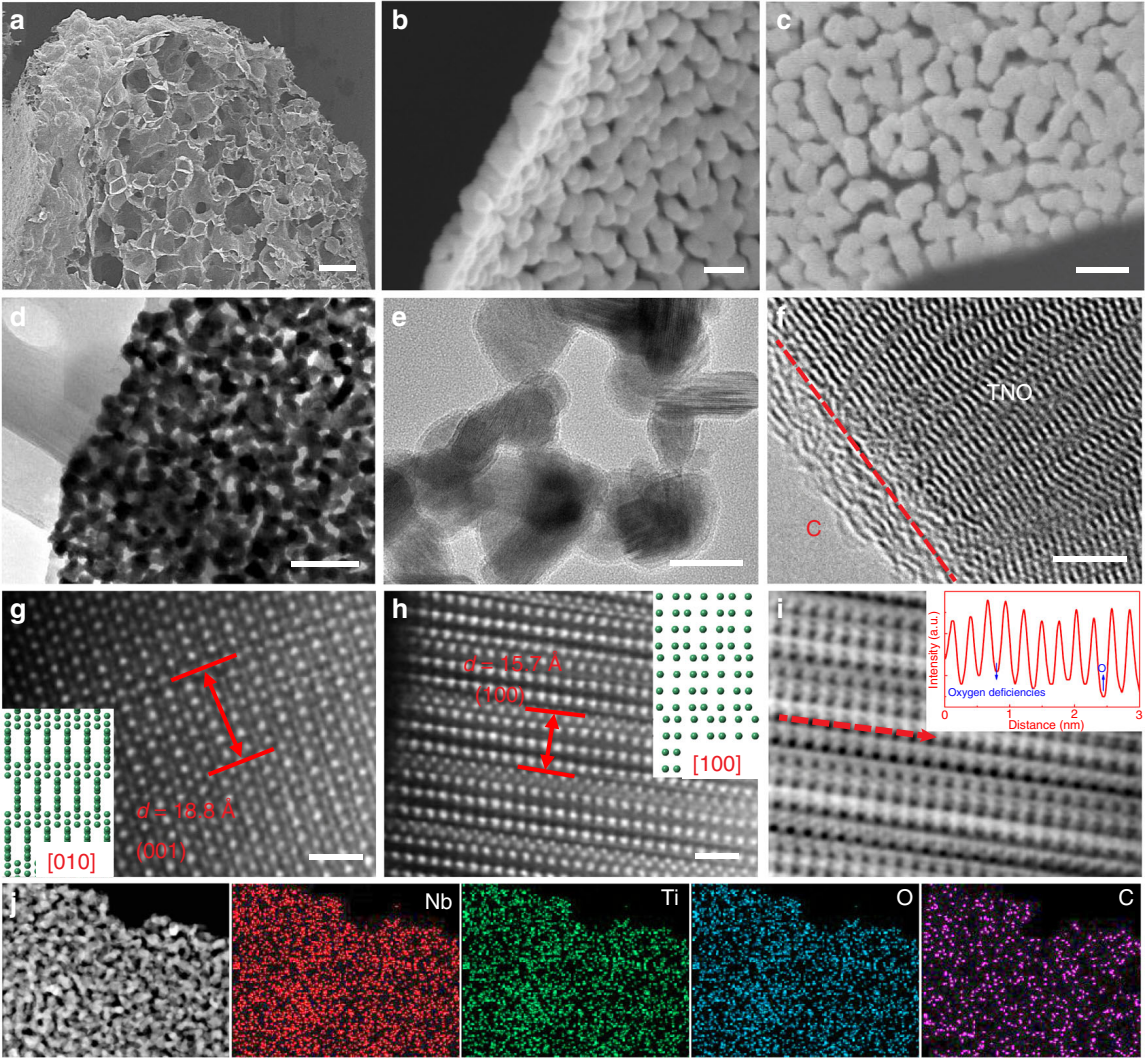
Synthesis and characterization of all products. **a** SEM images of hierarchical porous TNO_−*x*_@C_3_. **b** TNO_−*x*_@C_3_ nanosheets. **c** TNO_−*x*_@C_3_ from top view. **d** TEM images of TNO_−*x*_@C_3_. **e** TEM images of TNO_−*x*_@C_3_. **f** HRTEM images of TNO_−*x*_@C_3_. **g** HAADF images of TNO_−*x*_@C_3_. **h** HAADF images of TNO_−*x*_@C_3_. **i** ABF images of TNO_−*x*_@C_3_. **j** EDS elemental mapping images of Nb, Ti, O, and C in the TNO_−*x*_@C_3_ composite. Scale bars: **a** 200 μm, **b**, **c** 100 nm, **d** 200 nm, **e** 50 nm, **f** 5 nm, and **g**, **h** 1 nm.

As shown in Supplementary Fig. 1–2, puffed rice carbon (PRC) was employed as sacrificial template to obtain three dimensional (3D) hierarchical porous TNO network. A 3D hierarchical microcellular porous morphology was observed for the PRC, which consisted of interconnected secondary carbon sheets^18^. After solvothermal process and annealing in air, the sacrificial PRC template was decomposed and left a 3D hierarchical porous TNO network composed of cross-linked secondary nanosheets and tertiary nanoparticles with an average grain size of 50 nm and pore size of 10–50 nm (Supplementary Fig. 3). Followed by the controllable acetylene decomposition, the TNO primary grains were uniformly coated with carbon shells, forming 3D hierarchical porous Ti_2_Nb_10_O_29−*x*_@C (TNO_−*x*_@C) composites. The thickness of the boundary carbon could be controlled by the reaction temperature. In our case, three kinds of carbon thicknesses (1 nm-350 °C; 3 nm-400 °C; 5 nm-450 °C) were realized in the TNO_−*x*_@C composites and referred as TNO_−*x*_@C_1_, TNO_−*x*_@C_3_, and TNO_−*x*_@C_5,_ respectively (Fig. 1a–c and Supplementary Fig. 4). Note that the porous architectures are still well preserved in all the TNO_−*x*_@C composites, indicating that the boundary-coated carbon from the acetylene decomposition makes no contribution to the overall morphology.

**Fig. 2:**
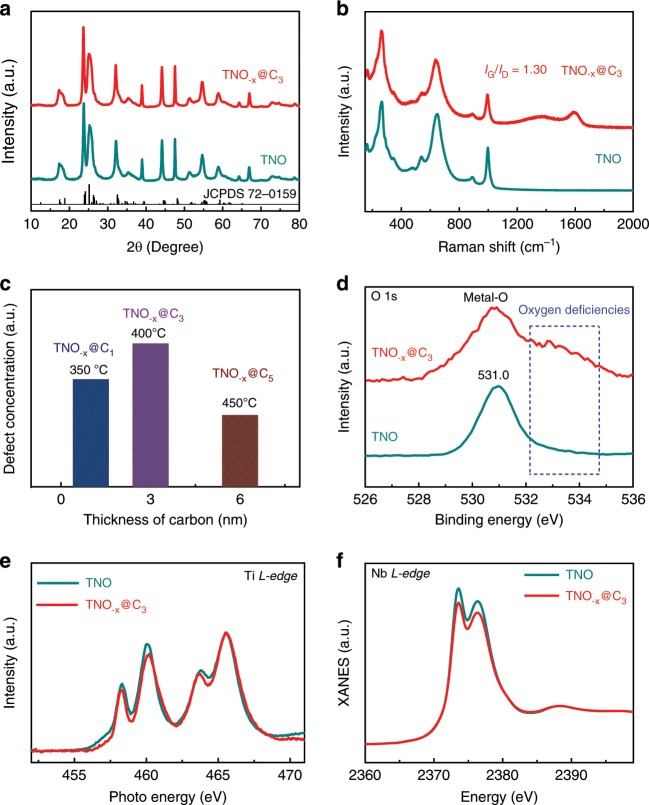
Phase and composition characterization of TNO and TNO_−*x*_@C_3_. **a** XRD patterns. **b** Raman spectra. **c** The relationship of defect concentration and thickness of carbon. **d** XPS spectra of O 1 s. **e** XANES spectra of Ti *L*-edge. **f** XANES spectra of Nb *L*-edge.

The microstructure evolutions of the products at different stages were investigated by transmission electron microscope (TEM). Consistent with the scanning electron microscope (SEM) results, the pure porous TNO nanosheets are composed of interconnected TNO nanograins of 50–60 nm with pore size of 10–50 nm (Supplementary Fig. 5a–b). The as-prepared TNO nanosheets are highly crystalline, as revealed by the high-resolution TEM (HRTEM) images (Supplementary Fig. 5c) as well as the selected area electron diffraction (SAED, Supplementary Fig. 5c inset) pattern. After controllable acetylene decomposition, interestingly, each primary TNO grain was homogeneously decorated with high-quality carbon layer with a thickness of ~3 nm produced at 400 °C (Fig. 1d–f). Similarly, uniform carbon coating was also achieved in the TNO_−*x*_@C_1_ and TNO_−*x*_@C_5_ composites (Supplementary Fig. 5d–i). In the light of similar morphology, the TNO_−*x*_@C_3_ composites are selected for the further discussion. The fine microstructure of TNO_−*x*_@C_3_ composites was investigated using scanning TEM (STEM) equipped with high-angle annular dark-field (HAADF) and annular bright-field (ABF) detectors. In the HAADF images (Fig. 1g, h), the atomic arrangements are represented with bright spots, in which the Ti/Nb sites are much brighter than the O sites due to the stronger scattering capability of electrons for high-*Z* atoms. Interplanar *d*-spacings of 18.8 Å and 15.7 Å corresponding to (001) and (100) planes are observed, demonstrating the presence of monoclinal Ti_2_Nb_10_O_29_ phase (JCPDS 72-0159) (Fig. 1g, h)^3,19,20^. It is known that the contrast of ABF image is highly sensitive to the light elements like O. Faint dark spots are visible at the O sites with fluctuant contrast, which was revealed by the inconsistent peak valley of the line profile (Fig. 1i). In addition, the weakening of the oxygen contrast firmly proves the abundant oxygen vacancies in the TNO_−*x*_@C_3_^21^. The introduction of the oxygen vacancies at TNO_−*x*_ could be due to the reduction of TNO by H_2_, which is generated from the decomposition of acetylene (C_2_H_2_): C_2_H_2_ = 2C + H_2_. In other words, the acetylene plays two important roles in the formation of TNO_−*x*_@C_3_ composites. On the one hand, the acetylene acts as the boundary carbon source, which deposits outside the primary grains of TNO during the decomposition process. On the other hand, the concomitant H_2_ could reduce TNO to produce the oxygen vacancy resulting in defective TNO_−*x*_. Such dual effects arising from the acetylene decomposition is unique and cannot be realized by the previous carbon-coating technologies. The uniformity of TNO_−*x*_@C_3_ was also verified by energy dispersive spectrometer (EDS) mapping. The elements of Ti, Nb, O, and C homogeneously distribute in the TNO_−*x*_@C_3_ sample (Fig. 1j), indicating the successful synthesis of TNO_−*x*_@C_3_ composites. It is noteworthy that our developed synergistic engineering of carbon and deficiency can be highly applicable to other metal oxides with tunable morphology, composition, and structure including TNO_−*x*_@C microspheres (Supplementary Fig. 6–7), Nb_2_O_5−*x*_@C microrods, TiO_2−*x*_@C nanoparticles, and ZnO_−*x*_@C nanorods (Supplementary Fig. 8). In a nutshell, the newly developed engineering of carbon and deficiency is a powerful method to simultaneously implement the conformal coating of boundary carbon layer and introduction of abundant oxygen deficiencies in various metal oxides. The phase evolution was monitored by X-ray diffraction (XRD) tests (Fig. 2a and Supplementary Fig. 9a). The diffraction peaks of all the samples could be well indexed to the Ti_2_Nb_10_O_29_ phase (JCPDS 72-0159)^22^, suggesting that the coating of boundary carbon does not affect the phase purity of TNO. Raman measurements were conducted to confirm the existence of the carbon-coating layer (Fig. 2b and Supplementary Fig. 9b). For the pure unmodified TNO nanosheets, the characteristic peaks at 264, 649, 894, and 1003 cm^−1^ are clearly recognized, matching well with the Ti_2_Nb_10_O_29_ phase. As for the TNO_−*x*_@C_3_ sample, apart from the above characteristic peaks of TNO, two newly emerged Raman peaks at 1352 cm^−1^ (disorder induced D-band) and 1585 cm^−1^ (a graphitic G-band) are detected and the intensity ratio of G-band to D-band (*I*_G_/*I*_D_) was about 1.30, suggesting the existence of carbon layer and its high crystallinity. Moreover, the *I*_G_/*I*_D_ ratio of TNO_−*x*_@C_3_ sample was higher than those of TNO_−*x*_@C_1_ and TNO_−*x*_@C_5_ samples, indicating the optimal crystallinity of carbon in the TNO_−*x*_@C_3_ sample. Thermogravimetric (TG) analysis (Supplementary Fig. 9c) demonstrates that the contents of carbon in the TNO_−*x*_@C_1_, TNO_−*x*_@C_3_, and TNO_−*x*_@C_5_ composites are 2.3, 3.2, and 6.8 wt.%, respectively, implying the controllable carbon coating by acetylene decomposition.

To study the oxygen deficiencies of the samples, electron paramagnetic resonance (EPR) tests were carried out. The TNO_−*x*_@C_3_ shows the strongest EPR signal at *g* = 2.0 (typical signal of oxygen vacancies) (Supplementary Fig. 9d), higher than those of TNO_−*x*_@C_1_ and TNO_−*x*_@C_5_ counterparts, while the pure TNO nanosheet sample only displays a flat line without obvious oxygen vacancy^23,24^. These results clearly confirm the presence of abundant oxygen deficiencies in TNO_−*x*_@C_3_. The TNO_−*x*_@C_3_ was also chosen as a representative to study its physicochemical property based on the optimized carbon layer and oxygen deficiency (Fig. 2c). As observed from X-ray photoelectron spectra (XPS, Fig. 2d), the O 1 s spectra of pure TNO nanosheets only presents an individual peak at 531.0 eV, belonging to metal-O bonding^8,23,24^. However, apart from the metal-O bonding, the TNO_−*x*_@C_3_ nanosheets also exhibit another peak characteristic of metal-OH bonding (532.6 eV), further indicating the existence of oxygen deficiencies in TNO_−*x*_@C_3_^8,23,24^. Meanwhile, the valence state change of Ti and Nb caused by oxygen deficiency is also proven by the Ti 2p and Nb 3d XPS spectra (Supplementary Fig. 9e–f)^25–27^. It is seen that, for the pure TNO nanosheets, two core levels of Ti 2p are assigned to Ti 2p_1/2_ (465.1 eV) and Ti 2p_3/2_ (459.4 eV), respectively. While the Ti peaks of TNO_−*x*_@C_3_ shift left as compared with the pure TNO nanosheets, demonstrating the existence of Ti^3+^ in the TNO_−*x*_@C_3_ (Supplementary Fig. 9e). In the meantime, Nb 3d spectra also show two characteristic core levels for the pure TNO nanosheets, indexed well into Nb 3d_5/2_ (207.6 eV) and Nb 3d_3/2_ (210.4 eV), respectively. While lower binding-energy centered at 210.1 eV (Nb 3d_3/2_) and 207.5 eV (Nb 3d_5/2_) are noticed for the TNO_−*x*_@C_3_ composite, demonstrating more Nb^4+^ ions in the TNO_−*x*_@C_3_ sample (Supplementary Fig. 9f). In short, the presence of Ti^3+^ and Nb^4+^ further verifies the presence of oxygen deficiency induced by such a carbon engineering strategy.

**Fig. 3:**
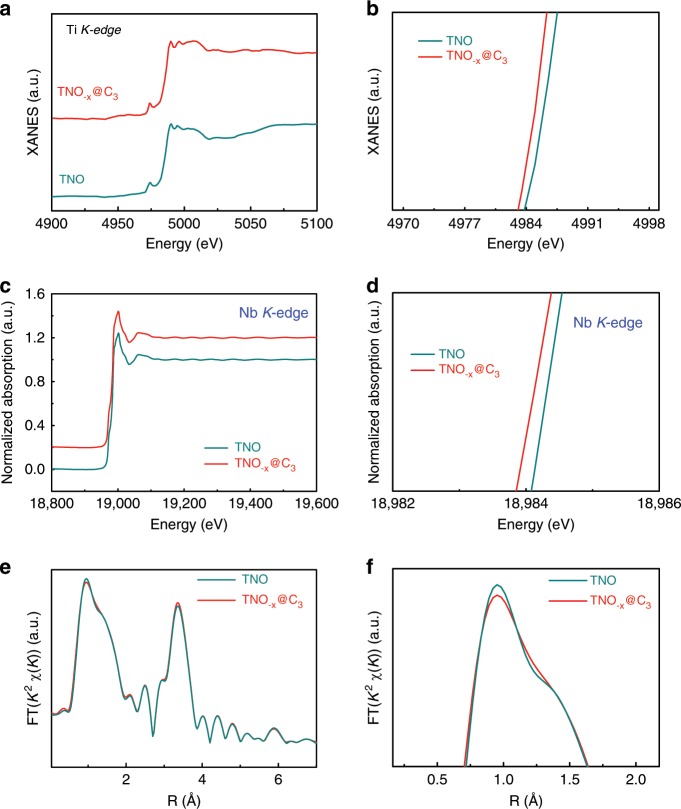
XANES and XAFS spectra of pure TNO and TNO_−*x*_@C_3_ samples. **a** Ti *K*-edge spectra. **b** Enlarged Ti *K*-edge spectra. **c** Nb *K*-edge spectra. **d** Enlarged Nb *K*-edge spectra. **e** Fourier transform of the *k*^2^-weighted Nb *K*-edge of the EXAFS spectra. **f** Fourier transform of the *k*^2^-weighted Nb *K*-edge of the enlarged EXAFS spectra.

In addition, X-ray absorption near-edge structure (XANES) analysis was employed to investigate the defective chemistry. Figure 2e shows the Ti *L*-edge XANES spectra, where four peaks centered at maxima over the energy range belong to *L*3_t2g_, *L*3_eg_, *L*2_t2g_, and *L*2_eg_, respectively. The lowered peak intensity of *t*_*2g*_ in TNO_−*x*_@C_3_ indicates that the Ti in TNO_−*x*_@C_3_ sample has a lower valence state due to partially occupied electronic states of *e*_*g*_ and *t*_*2g*_ at the surface region. The above difference is attributed to the presence of oxygen deficiencies, which can cause loss of long-range order^28^. Meanwhile, Fig. 2f presents the normalized Nb *L*_2_ and *L*_3_-edge XANES absorption spectra of the two samples, matching well with the transition of 2*p*1/2→nd and 2*p*3/2→nd, respectively. Notice that the TNO_−*x*_@C_3_ shows lower peak intensity, suggesting a number decrease of Nb 3d holes as a result of the generated oxygen deficiencies^23,29^. Moreover, as shown in Fig. 3a, b, the Ti *K*-edge spectra of TNO_−*x*_@C_3_ exhibits similar characteristic compared with the TNO phase. Nevertheless, the TNO_−*x*_@C_3_ sample displays lower valance state according to left shift of absorption edge position, implying the presence of abundant oxygen deficiencies. In order to deepen the understanding of the electronic state and oxygen deficiencies, XAFS spectra of Nb *K*-edge were tested for both of the samples. The XANES of the TNO_−*x*_@C_3_ shows a smaller energy of the absorption edge (Fig. 3c, d), consistent with the XPS results. Figure 3e, f exhibits the corresponding *R* space curves of *k*^2^ [*χ* (*k*)] weighted Fourier transform spectra^30,31^. The peaks centered at 0–2 Å in these TNO samples belong to Nb–O bond, and another peak at 3–4 Å is attributed to Nb–Nb or Nb–Ti vectors^23,32^. It is worth noting that the TNO_−*x*_@C_3_ displays lower intensity in the Nb–O bond, indicating the lower oxygen coordination number and more oxygen deficiencies. The above results support each other to demonstrate that such boundary carbon engineering could not only provide scalable carbon layer, but also introduce oxygen deficiencies in the TNO.

### First-principle calculations of TNO

**Fig. 4:**
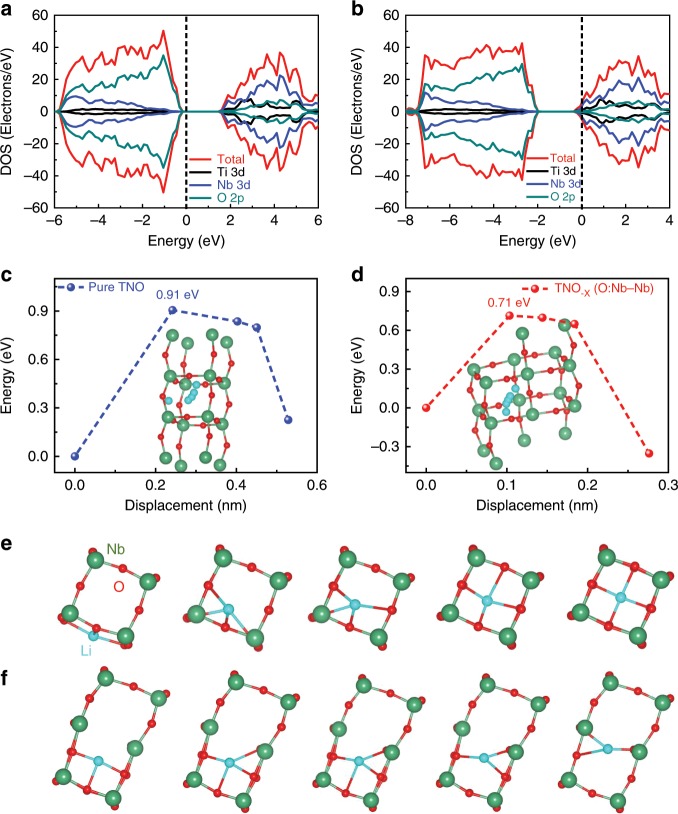
Density of states (DOS) of pure TNO and defective TNO_−*x*_. **a** DOS of pure TNO. **b** DOS of defective TNO_−*x*_ (O:Ti–Nb). **c** The Li ion diffusion energy barrier for TNO. **d** The Li ion diffusion energy barrier for TNO_−*x*_(O:Nb–Nb). **e** The Li ion diffusion pathways for TNO. **f** The Li ion diffusion pathways for TNO_−*x*_(O:Nb–Nb).

To illustrate the influence of oxygen vacancy on the properties of TNO, the first-principle calculations were employed. As shown in Supplementary Fig. 10, there are five main cases of oxygen vacancies: oxygen vacancy between Ti and Nb atoms (TNO_−*x*_ (O:Ti–Nb)), between Nb and Nb atoms (TNO_−*x*_ (O:Nb–Nb)), between Nb, Nb, and Nb atoms (TNO_−*x*_ (O:Nb–Nb–Nb)), between Nb, Ti, and Nb atoms (TNO_−*x*_ (O:Nb–Ti–Nb)), between Ti, Nb, and Ti atoms (TNO_−*x*_ (O:Ti–Nb–Ti)). Their crystal structures are optimized and the corresponding lattice parameters and energies are listed in Supplementary Table 1. Note that the TNO_−*x*_ (O:Nb–Nb) has relatively low energies than the others, demonstrating that the TNO_−*x*_ (O:Nb–Nb) is more stable and the oxygen atom is prone to lose at the position. Moreover, the total density of states and partial density of states were calculated (Fig. 4a, b and Supplementary Fig. 11). For the valence band of the perfect TNO, it is found that the strong hybridizations between Ni3*d*-O2*p* and Ti3*d*-O2*p* are revealed by their obvious overlaps and consistent amplitudes^33^. Impressively, the electron conductivity can also be significantly affected by oxygen vacancy. For the defective TNO_−*x*_ (O:Ti–Nb) and TNO_−*x*_ (O:Nb–Nb), the Fermi level transforms from the top of the valence band of the perfect crystal to the bottom of the conduction band of defective TNO_−*x*_, which can greatly improve the intrinsic electronic conductivity of electrode material. For the defective TNO_−*x*_ (O:Nb–Nb–Nb), TNO_−*x*_ (O:Nb–Ti–Nb), and TNO_−*x*_ (O:Ti–Nb–Ti), not only the Fermi level moves toward high-energy direction, but also the impurity levels appear between the valence and conduction band. And the composition of the impurity level is heavily dependent on the oxygen vacancy position. Due to the existence of the impurity levels, the electronic conductivity of the defective TNO_−*x*_ can be greatly enhanced. In addition, it could be seen that the partial charge density of the perfect crystal has a strong locality near the Fermi plane, while the partial charge density of the crystal with oxygen vacancy is relatively dispersed. This further indicates that oxygen deficiency can improve electrical conductivity (Supplementary Fig. 12). Moreover, the Li ion diffusion pathways and energy barrier for TNO and TNO_−*x*_ (O:Nb–Nb) were further explored using nudged elastic-band (NEB) method. Herein, the defective TNO_−*x*_ (O:Nb–Nb) was chosen as a case due to lowest formation energy. For simplification, the smallest crystal structure unit was used in the NEB calculation process. As shown in Fig. 4e, f, the Li ion diffusion pathways of TNO and TNO_−*x*_ (O:Nb–Nb) are described, respectively. The initial site of Li ion is the lowest energy oxygen deficiency site and the final is the lowest energy interstitial site. Their corresponding energy barriers are shown in Fig. 4c, d. It could be seen that the energy barrier of the pure TNO is only 0.91 eV, while the energy barrier of the TNO_−*x*_ (O:Nb–Nb) reduces to 0.71 eV. This indicates that the Li ion is easier to migrate in defective TNO_−*x*_ (O:Nb–Nb) than the pure TNO. Based on the above results, enhanced electrical conductivity and ionic transfer are ensured for the TNO_−*x*_@C_3_ composites and better Li ion storage performance can be anticipated.

### Electrochemical performance of TNO_−*x*_@C_3_ composites

**Fig. 5:**
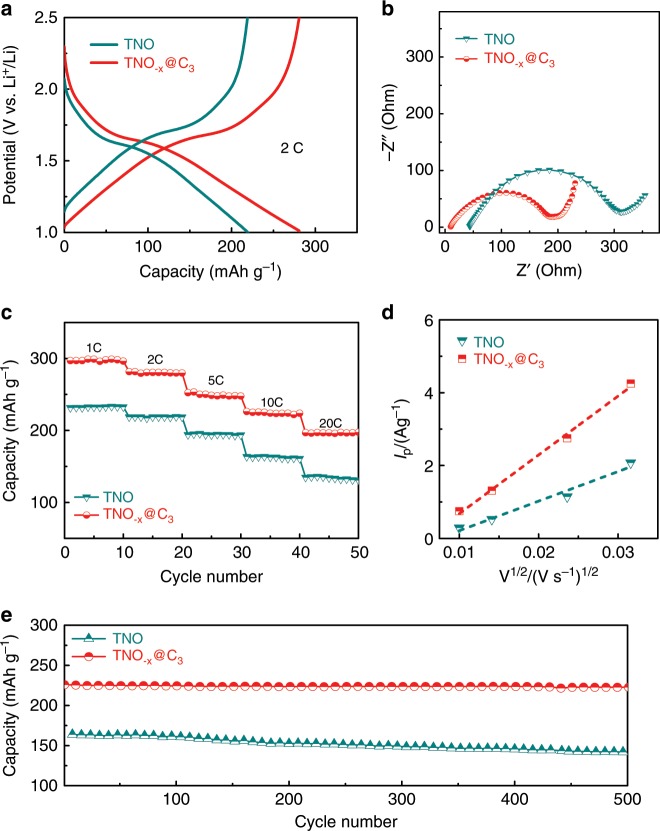
Electrochemical characterization of TNO and TNO_−*x*_@C_3_ electrodes. **a** Discharge/charge profiles. **b** Nyquist plots at the fully discharge state at the first cycle. **c** Rate capability. **d** Peak current I_p_ as a function of square root of scan rate V^1/2^. **e** Cycling stability at 10 C.

The electrochemical performance was firstly monitored by charge–discharge tests in the voltage window of 1.0–2.5 V (Fig. 5a). The discharge specific capacity of TNO_−*x*_@C_3_ is 280 mA h g^−1^, higher than that of pure TNO electrode (217 mA h g^−1^). The improved electrochemical performance was also verified by electrochemical impedance spectrum (EIS) test. The TNO_−*x*_@C_3_ electrode presents smaller charge-transfer resistance and ohmic resistance (Fig. 5b), indicating that the oxygen deficiencies and carbon layer can facilitate charge transfer and boost reaction kinetics. Apparently, the TNO_−*x*_@C_3_ electrode exhibits superior high-rate performance with large capacities of 253 mA h g^−1^, 226 mA h g^−1^, and 197 mA h g^−1^ at 5 C, 10 C, and 20 C (Fig. 5c), respectively, superior to those of the TNO electrode (195 mA h g^−1^ at 5 C, 162 mA h g^−1^ at 10 C, and 136 mA h g^−1^ at 20 C) (Supplementary Fig. 13c). Note that the introduction of abundant oxygen deficiencies and high-quality carbon coating can enhance electrochemical rate performance because of greatly improved intrinsic conductivity and integrated conductive network. Meanwhile, the obtained *I*_p_ (intensity of Nb^4+^/Nb^5+^ anodic peak) is plotted as a function with V^1/2^ (V is the scan rates) in Fig. 5d and Supplementary Fig. 13a–b, whose slope stands for the Li^+^ diffusion coefficient. Obviously, the TNO_−*x*_@C_3_ electrode displays a steep slope with 1.98 times larger than that of the pure TNO electrode, suggesting larger Li^+^ diffusion coefficient. More importantly, long-term durability is achieved for the TNO_−*x*_@C_3_ electrode (Fig. 5e), which displays a discharge capacity of 223 mA h g^−1^ at 10 C with a capacity retention of 98.7% over 500 cycles. In contrast, the TNO counterpart only shows a capacity of 140 mA h g^−1^ (capacity retention of 85.9% over 500 cycles). The enhanced rate performance and long cycle life of TNO_−*x*_@C_3_ electrode arise from the synergistic effects between the oxygen deficiencies existence and controllable boundary carbon coating.

## Discussion

**Fig. 6:**
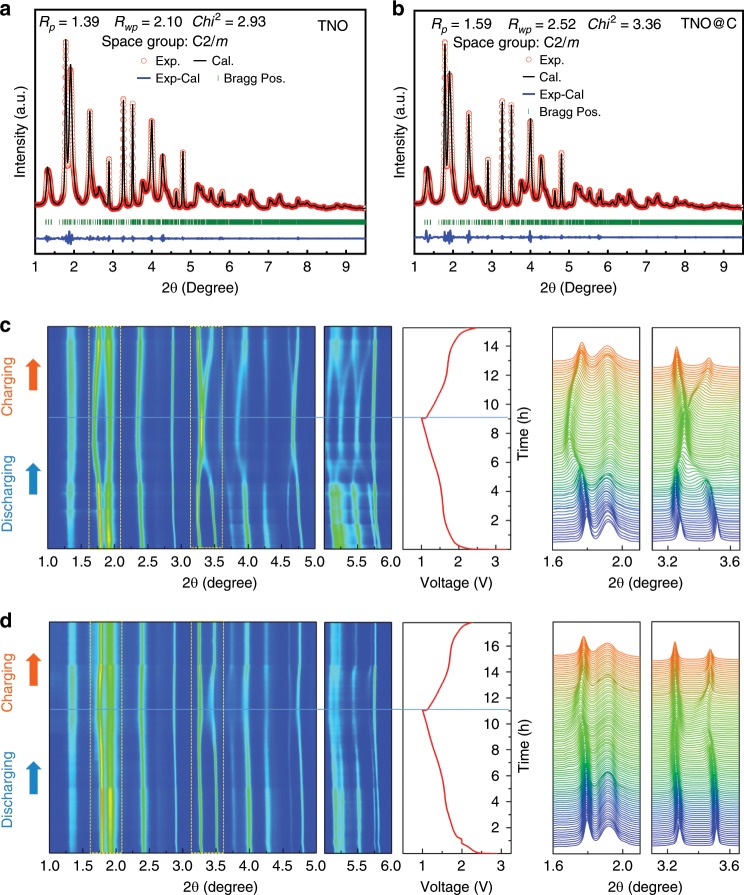
Synchrotron HRXRD characterization of TNO and TNO_−*x*_@C_3_. **a** Ex situ synchrotron HRXRD characterization of TNO. **b** Ex situ synchrotron HRXRD characterization of TNO_−*x*_@C_3_. **c** In situ synchrotron HEXRD characterization voltage profile and corresponding contour plot of the XRD pattern evolution of TNO during the first charge–discharge process. **d** In situ synchrotron HEXRD characterization voltage profile and corresponding contour plot of the XRD pattern evolution of TNO_−*x*_@C_3_ during the first charge–discharge process. (Contour plot of the (011), (400), (317), and (700) peaks evolution).

**Fig. 7:**
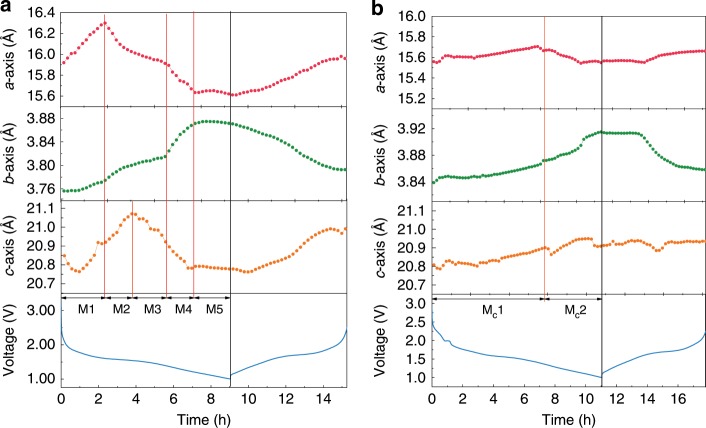
The voltage profile and the lattice parameter evolution process. **a** pure TNO. **b** TNO_−*x*_@C_3_.

In addition, a deeper understanding of superior lithium storage performance by synergistic engineering of carbon and deficiency was investigated through ex situ HEXRD to detect the lattice parameters of the pure and TNO_−*x*_@C_3_ (Fig. 6a, b). The nanoscale nature of both materials gives rise to a significant line broadening of the diffraction peaks, therefore Lebail method with size model of spherical harmonics was adopted for the refinements of the XRD patterns. All the Bragg peaks could be successfully described with the monoclinic ReO_3_ shear structure (space group, C2/m), indicating that the carbon will not change the long-range coherence of TNO framework^4^. Nevertheless, a remarkable lattice expansion is revealed for the TNO_−*x*_@C_3_ sample in contrast with the pure TNO (Supplementary Table 2). This could be ascribed to the existence of Ti^3+^ and Nb^4+^ (with larger ion-radiuses than Ti^4+^ and Nb^5+^) that are reduced by the oxygen vacancies^7,34^. The increased lattice parameters of TNO_−*x*_@C_3_ might further indicate the probable missing location of lattice oxygen is in the Nb–Nb site (TNO_−*x*_ (O:Nb–Nb)) according to the consistent results of HEXRD and Density functional theory (DFT) calculations. The lattice expansion further suggests that the TNO_−*x*_@C_3_ electrode is beneficial for Li ions transfer. In the meantime, in situ synchrotron HEXRD measurements were also performed on both pure TNO and TNO_−*x*_@C_3_ within the first discharge–charge cycle. From the 2D contour plots of the XRD patterns (Fig. 6c, d), remarkably, a striking shift of all the Bragg peaks is observed for the pure TNO, while the peak shifting for the TNO_−*x*_@C_3_ is less obvious. In addition, the pure TNO patterns also show a more anisotropic feature in contrast with TNO_−*x*_@C_3_. Two typical groups of the peak profiles are presented to detail these behaviors. Accordingly, it could be speculated that the lattice of the TNO_−*x*_@C_3_ experiences a moderate structural change over cycling due to modulation of synergistic engineering of carbon and deficiency. The lattice evolutions of both materials are extracted from the in situ XRD patterns for a more specific view. For the pure TNO, four structural transitions occur continuously as the lithium ions embed into the framework, leading to five monoclinic structures (referred as M1–M5) over the whole discharging stage (Fig. 7a). These transitions are fingerprinted by the abruptly evolutive breaks of the axes, as it signals the potential barriers to be overcome^35,36^. In detail, the transitions from M1 to M4 (3.0–1.5 V) are irreversible, during which the lattice parameters exhibit a nonmonotonic dependence on lithium content. This period could be recognized as the activation process, i.e., the rearrangements of host-atoms to tolerate Li-insertion, which could inevitably induce mechanical fractures that deteriorate reversible capacity and cyclic kinetics. As for the TNO_−*x*_@C_3_, only one structural transition between two monoclinic structures (Mc1 and Mc2) is observed upon discharging, and just as importantly, the lattice anisotropy is also significantly weakened (Fig. 7b) compared with the pure TNO material. This provides evidence that the harmful activation of TNO could be efficiently inhibited through building these carbon-coated boundaries. In addition, the subsequent delithiation leads to a coupled expansion of *a*-/*c*-axis and an independent contraction of *b*-axis for both materials, indicating that the Li^+^ diffusion path could be along *b*-direction. Notably, an obvious weakening of the lattice changes along all directions is observed for the TNO_−*x*_@C_3_. For these two materials, this solid-solution reaction is almost reversible, so the weakening of the periodic structural fluctuation in the TNO_−*x*_@C_3_ could reduce the repeated lattice strain and eventually lead to a significant improvement on the cycling performance. To better understand the structural features of TNO_−*x*_@C_3_ electrodes, ex situ electrochemical dilatometry was employed. Supplementary Fig. 14 presents the electrode thickness variation of TNO_−*x*_@C_3_ and TNO during the first galvanostatic discharge/charge cycle according to the SEM results (Supplementary Fig. 15). Obviously, discharge and charge processes are accompanied with a thickness increase and then decrease for two electrodes, respectively. In agreement with the results of in situ XRD analysis above, the TNO_−*x*_@C_3_ electrode shows a lower expansion rate than TNO electrode, mainly due to the buffer function of boundary carbon coating. In all, from the structural point of view, the origin of the enhanced cycling performance of TNO_−*x*_@C_3_ is twofold: the inhibition of the activation process and the weakening of the cyclic structural change. This could be explained by the stability of the carbon-coated boundaries, which constrains the internal grains from complex phase transitions upon the initial lithiation and severe lattice change induced from the solid-solution reaction.

To summarize, we have verified a universal and controllable low-temperature engineering of carbon and deficiency down to primary-grain level to achieve fast charging/discharging electrode materials with conformal and thickness-adjustable boundary carbon. Impressively, relying on such exquisite boundary architecture, significantly improved electron transport and enlarged Li^+^ diffusion path, shortened activation process and better structural stability are readily realized in the electrode materials due to the regulation of oxygen vacancy and weakened lattice dynamical anisotropy. As a result, the enhanced high-rate energy storage performance has been triggered in the TNO_−*x*_@C_3_ electrodes, which show superior rate capability (197 mA h g^−1^ at 20 C, corresponding to 3 min charge time) and remarkable cycling durability in lithium ion storage, much better than other counterparts (Supplementary Table 3). Most importantly, our developed engineering of carbon and deficiency is versatile and demonstrated to be applicable in a series of metal oxides systems (TNO_−*x*_@C_,_ Nb_2_O_5−*x*_@C, TiO_2−*x*_@C, and ZnO_−*x*_@C) with adjustable structure, morphology, and composition. Our work reveals a ground-breaking method in the boundary carbon design/decoration of composite materials for electrochemical energy storage.

## Methods

### Synthesis of TNO_−*x*_@C_3_ composites

First, the 3D hierarchical porous pure TNO were fabricated via a facile solvothermal method. The puffed rice was annealed at 800 °C for 3 h in Ar to obtain PRC as the sacrificial carbon template. Then, 0.56 g Ti–(OC_3_H_7_)_4_ and 2.7 g NbCl_5_ were added into 60 mL ethanol to form the homogeneous solution after stirring. Then, the above solution was transferred into a Teflon-linked steel autoclave with the PRC immersed into it, and kept at 200 °C for 10 h. After that, the as-obtained materials were annealed in air at 800 °C for 2 h to form pure 3D hierarchical porous TNO. Finally, the 3D hierarchical porous TNO powder was annealed at 400 °C for 1 h in C_2_H_2_ atmosphere to obtain TNO_−*x*_@C_3_ composites. For comparison, the TNO_−*x*_@C_1_ and TNO_−*x*_@C_5_ composites were obtained by annealing at 350 °C and 450 °C for 1 h in C_2_H_2_ atmosphere, respectively.

### Samples characterizations

The morphologies and microstructures of all samples were monitored by SEM (Hitachi S-4800) and TEM-HRTEM (JEOL 2100F at 200 kV) and spherical aberration-corrected scanning transmission electron microscopy (JEOL 2100F). XRD (Rigaku D/MAX 2550/PC) patterns were recorded in the range of 10–80°. Raman spectra were measured under laser excitation at 514 nm (LabRam HR UV). TG measurement was carried out by using Netzsch STA 449C thermal analyser. The chemical components and valence state were conducted by XPS (eSCALAB250Xi). XANES spectra (Ti *K*-edge and Nb *L*-edge) were collected at the 4W7A beamline station in Beijing Synchrotron Radiation Facility (BSRF, operated at 2.5 GeV with a maximum current of 250 mA) of China. The spectra were performed in the total electron yield (TEY) mode and the sample drain current was collected with a base pressure under than 5 × 10^−8^ Pa. X-ray absorption fine structure (XAFS) spectra (Nb *K*-edge) were carried out at the BL14W1 beamline station in Shanghai Synchrotron Radiation Facility (SSRF, operated at 3.5 GeV with a maximum current of 230 mA), China. The synchrotron beamline was monochromatized with Si (111) and Si (311) double-crystal monochromators to reduce the harmonic component. The data were collected at room temperature in fluorescence mode and analyzed according to the standard procedures using the Demeter software packages (University of Chicago). XANES spectra (Ti *L*-edge) were measured at beamline station BL12B in National Synchrotron Radiation Laboratory (NSRL, operated at 800 MeV with a maximum current of 300 mA) of China. The samples were placed on double-sided carbon tape for X-ray spectroscopy and all spectra were obtained with the TEY mode^37^. The “a.u.” was the abbreviation for “arbitrary units”.

### Measurements of electrochemical performance

For the electrochemical performance evaluation, the working electrode was prepared by mixing TNO or TNO_−*x*_@C_3_, Super P conductive carbon, and polyvinylidene fluoride in an 80:10:10 weight ratio, the lithium foil and microporous polypropylene film (Celgard, 2300) were used as counter electrode and separator, respectively. The electrochemical properties were tested in CR2025-type coin cells using 1.0 M LiPF_6_ in acetylene carbonate/dimethyl carbonate (EC/DEC, 1:1, by volume) as the electrolyte. EIS and cyclic voltammetry (CV) curves were conducted on a CHI 660E electrochemical workstation (CH Instruments Inc., Shanghai). CV tests were carried out from 1.0 to 2.5 V at different scan rates. For EIS, the amplitude of the sine perturbation signal was 5 mV, and the frequency was scanned from the highest (100 kHz) to the lowest (10 mHz). The galvanostatic charge/discharge measurement was performed on a NEWARE battery testing system between cutoff voltages of 1.0–2.5 V (vs. Li/Li^+^) at different current densities at room temperature. The loading amount of active material was about 2 mg cm^−2^ (1C = 396 mA g^−1^).

### Collection and analysis of in situ synchrotron XRD

The in situ XRD measurements were performed using 11-ID-C beamline at Advanced Photon Source (APS) of Argonne National Laboratory, with the X-ray wavelength of 0.1173 Å. Si (113) single crystal was used as monochromator for an X-ray beam at 105.7 keV. In a typical in situ collection, the coin cell with TNO or TNO_−*x*_ anode was operated with a constant current of 0.075 C. A single XRD pattern was obtained using a time period of 120 s^35^. The 2D diffraction patterns were collected in the transmission geometry with a Perkin-Elmer detector. The sample-to-detector distance, detector tilt angles and the instrumental resolution function were calibrated with a standard sample of CeO_2_. The integration and calibration of the 2D patterns were performed with the Fit2D software, and the lattice parameters were extracted from the integrated XRD patterns using Fullprof software. For the Lebail method, the backgrounds were subtracted by linear interpolation and the peak shapes were described using Thompson–Cox–Hastings profile function to separate the Gaussian and Lorentzian contributions to the Bragg peaks^34,38^. An anisotropic Lorentzian size broadening (spherical harmonics) was employed to describe the microstructure of the nanoscale TNO materials.

### Calculations

DFT calculations were carried out to investigate the electronic structure by using the first-principle and projector-augmented wave^39^ as implemented in Vienna ab initio simulation package^40^. The spin-polarized generalized gradient approximation in the form of Perdew–Burke–Ernzerhof formulation^41^ was employed for the correlation functional and electronic exchange. The plane-wave energy cutoff was set to 500 eV. The convergence tolerances and Hellmann–Feynman force on each atom were 0.01 eV Å^−1^ and 10^−5^ eV for maximum, respectively. Brillouin-zone integration was modeled by a periodic scheme 3 × 3 × 3 *k*-point mesh, where the lattice vectors together with unit cell shape and size and atomic coordinates were relaxed^33^. The Li ion diffusion pathways calculations were employed using the climbing image nudged elastic-band method.

## Supplementary information

Supplementary Information

## Data Availability

The data that support the findings of this study are available from the authors upon reasonable request, see author contributions for specific datasets.
