# Supplementary material for: Boosting fast energy storage by synergistic engineering of carbon and deficiency
Source: Nat Commun. 2020 Jan 9;11:132. doi: 10.1038/s41467-019-13945-1 (PMC6952377; doi:10.1038/s41467-019-13945-1)
Supplement: Supplementary file 1 — Supplementary Information [file 41467_2019_13945_MOESM1_ESM.pdf]

Supplementary Information

Boosting fast energy storage by synergistic engineering of carbon and deficiency

Deng et al.

Supplementary Figures

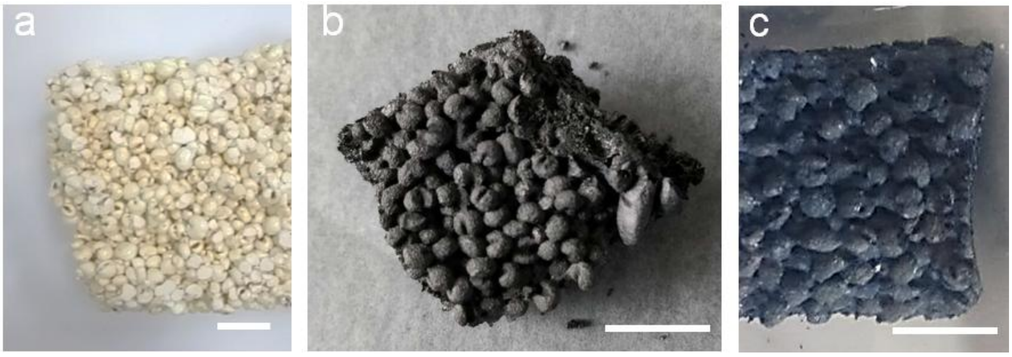

Supplementary Figure 1. Digital images. (a) PR. The scale bar is 1 cm. (b) PRC. The scale bar is 1 cm. (c) PRC/TNO precursor. The scale bar is 5 mm.

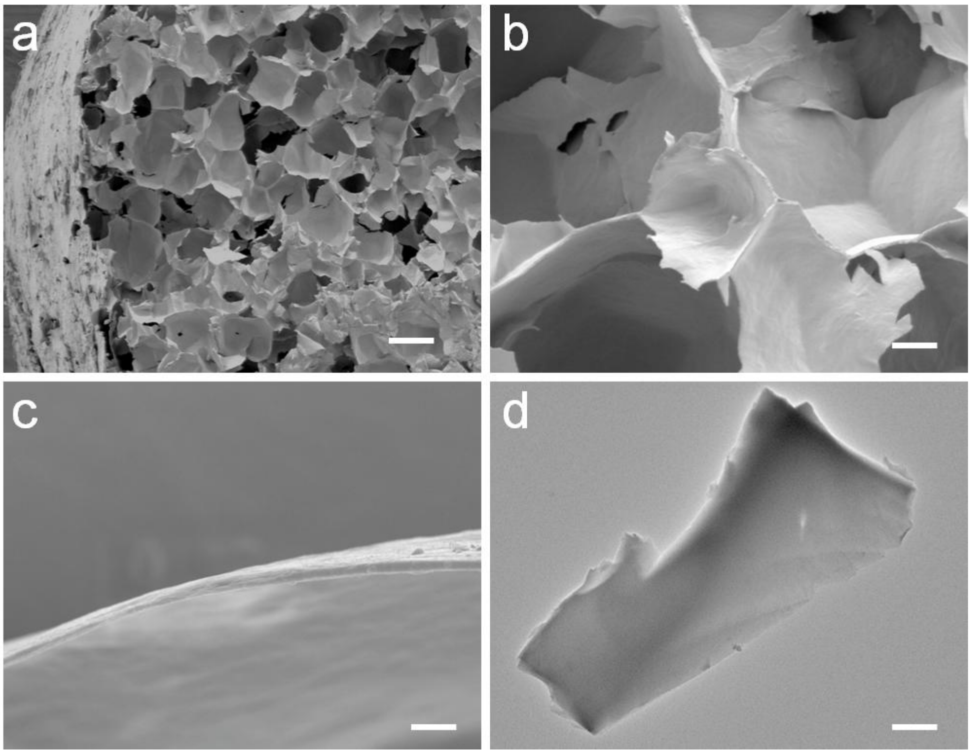

Supplementary Figure 2. Morphology characterization. (a-c) SEM images of PRC and (d) TEM image of PRC. Scale bars: (a) 100 μm, (b) 20 μm, (c) 5 μm and (d) 500 nm.

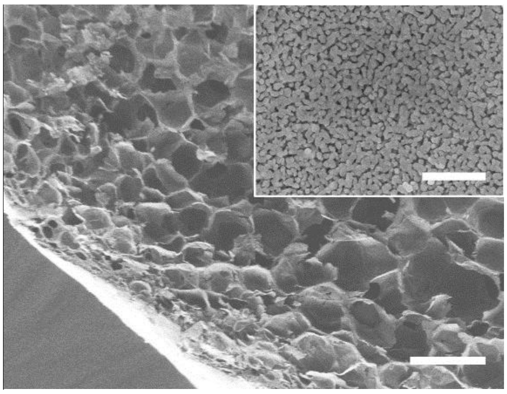

Supplementary Figure 3. SEM image of pure TNO. The scale bar is 100 μm. (Enlarged SEM image of pure TNO in inset. The scale bar is 500 nm.)

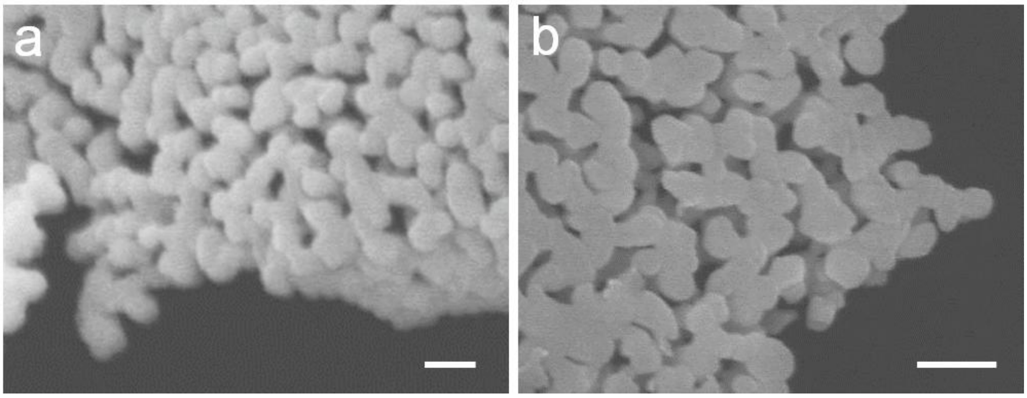

Supplementary Figure 4. Morphology characterization. (a) SEM image of $TNO_{-x}@C_1$. The scale bar is 100 nm. (b) $TNO_{-x}@C_5$. The scale bar is 100 nm.

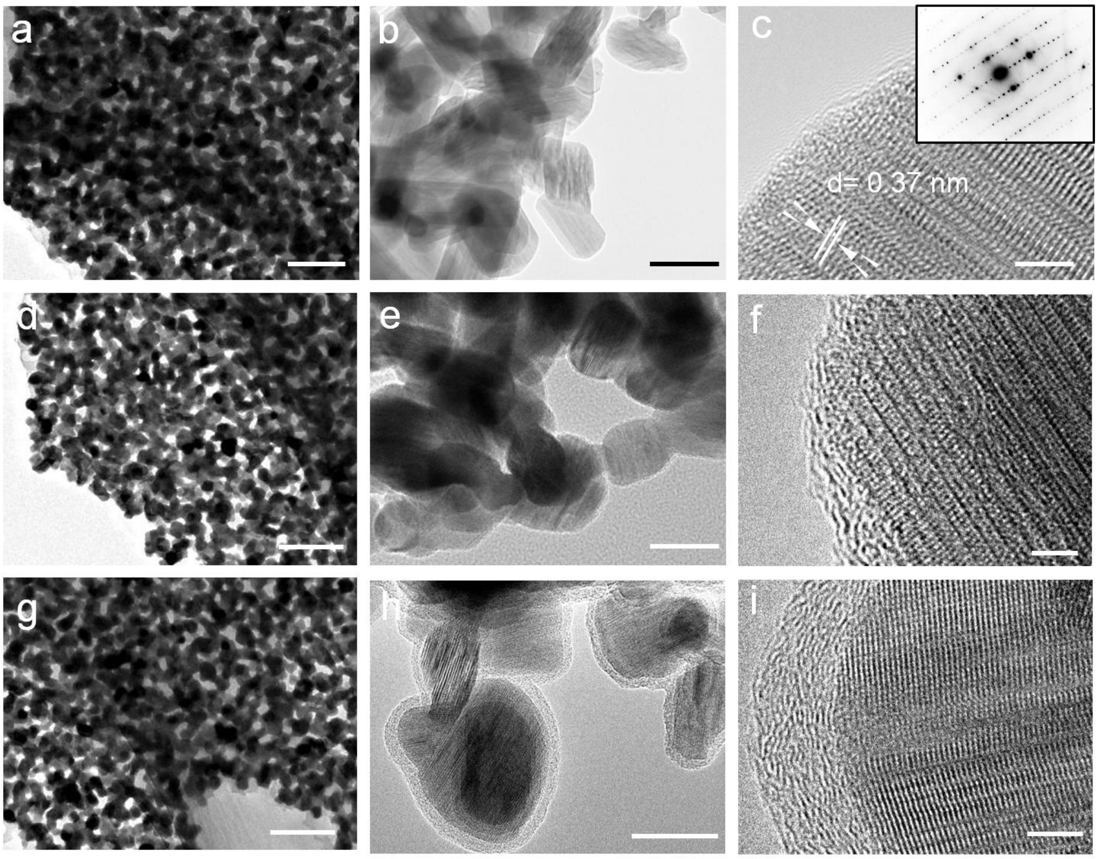

Supplementary Figure 5. Transmission electron microscopy characterization. (a-c) TEM-HRTEM image of pure TNO nanosheets. (d-f) TEM-HRTEM image of $TNO_{-x}@C_1$ nanosheets. (g-i) TEM-HRTEM image of $TNO_{-x}@C_5$ nanosheets. Scale bars: (a, d, g) 200 nm, (b, e, h) 50 nm and (c, f, i) 5 nm.

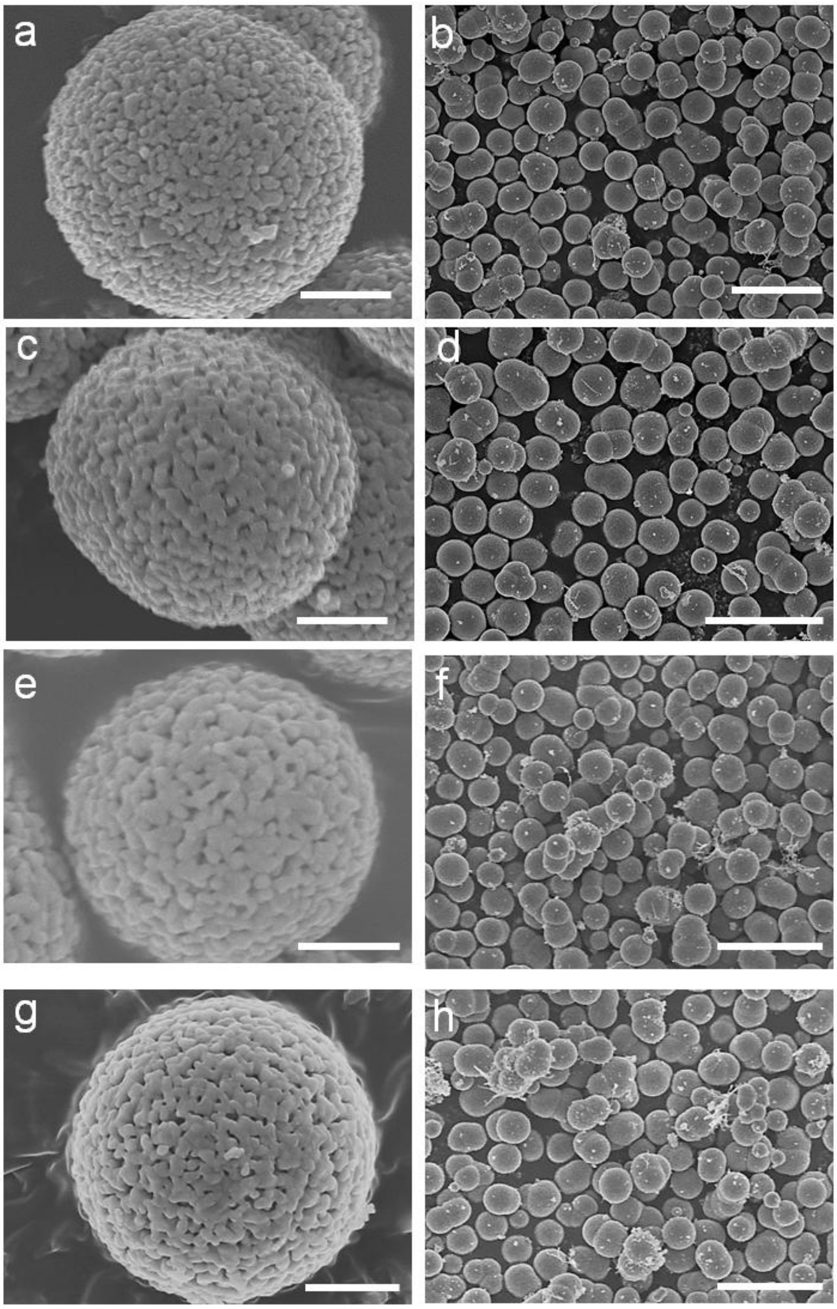

Supplementary Figure 6. Morphology characterization. (a-b) SEM images of TNO microspheres. (c-d) SEM images of $TNO_{-x}@C_1$ microspheres. (e-f) SEM images of $TNO_{-x}@C_3$ microspheres. (g-h) SEM images of $TNO_{-x}@C_5$ microspheres. Scale bars: (a, c, e, g) 500 nm and (b, d, f, h) 5 μm.

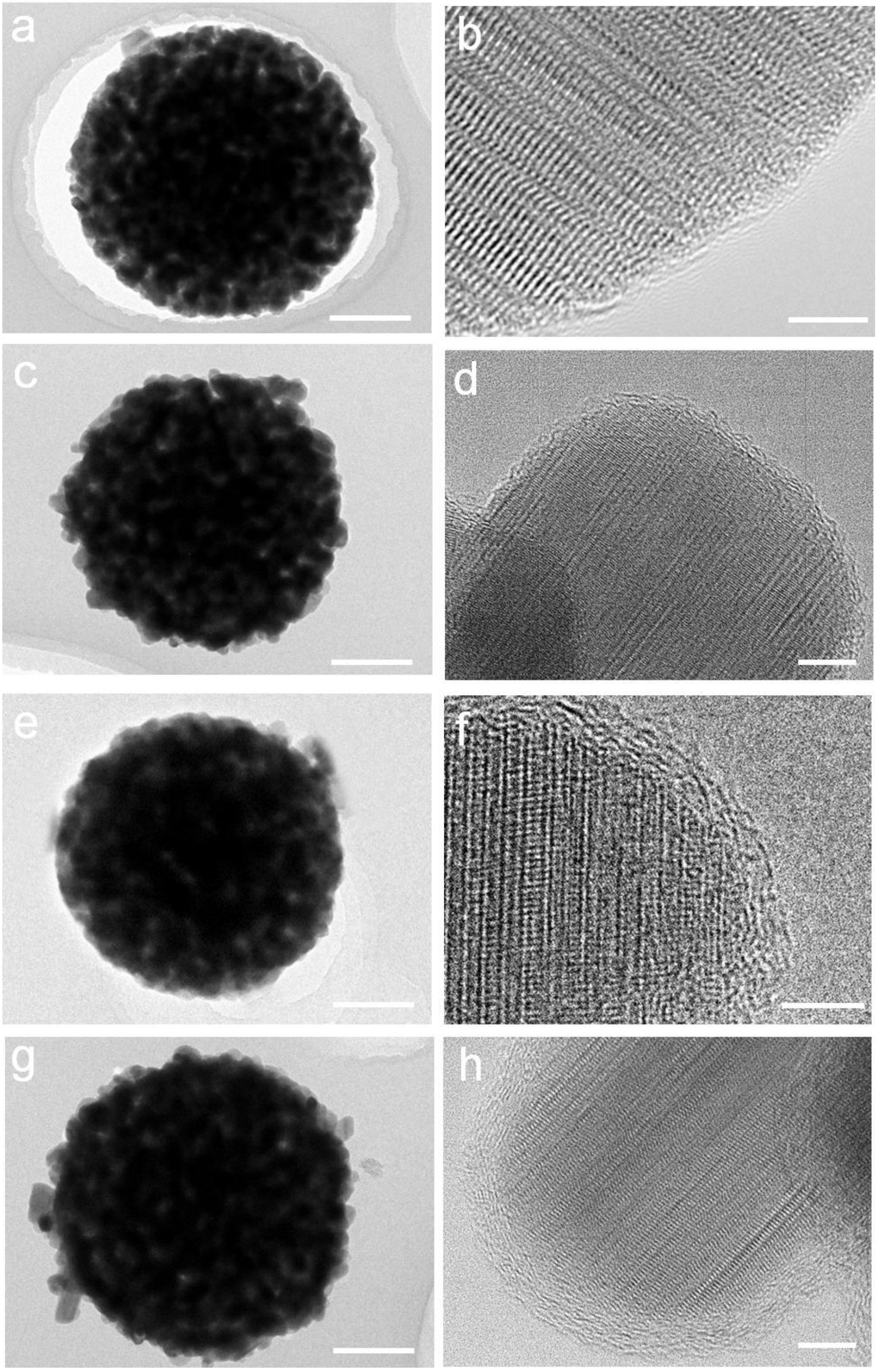

Supplementary Figure 7. Transmission electron microscopy analysis of microspheres. (a-b) TEM-HRTEM images of TNO microspheres. (c-d) TEM-HRTEM images of $TNO_{-x}@C_1$ microspheres. (e-f) TEM-HRTEM images of $TNO_{-x}@C_3$ microspheres. (g-h) TEM-HRTEM images of $TNO_{-x}@C_5$ microspheres. Scale bars: (a, c, e, g) 500 nm and (b, d, f, h) 5 nm.

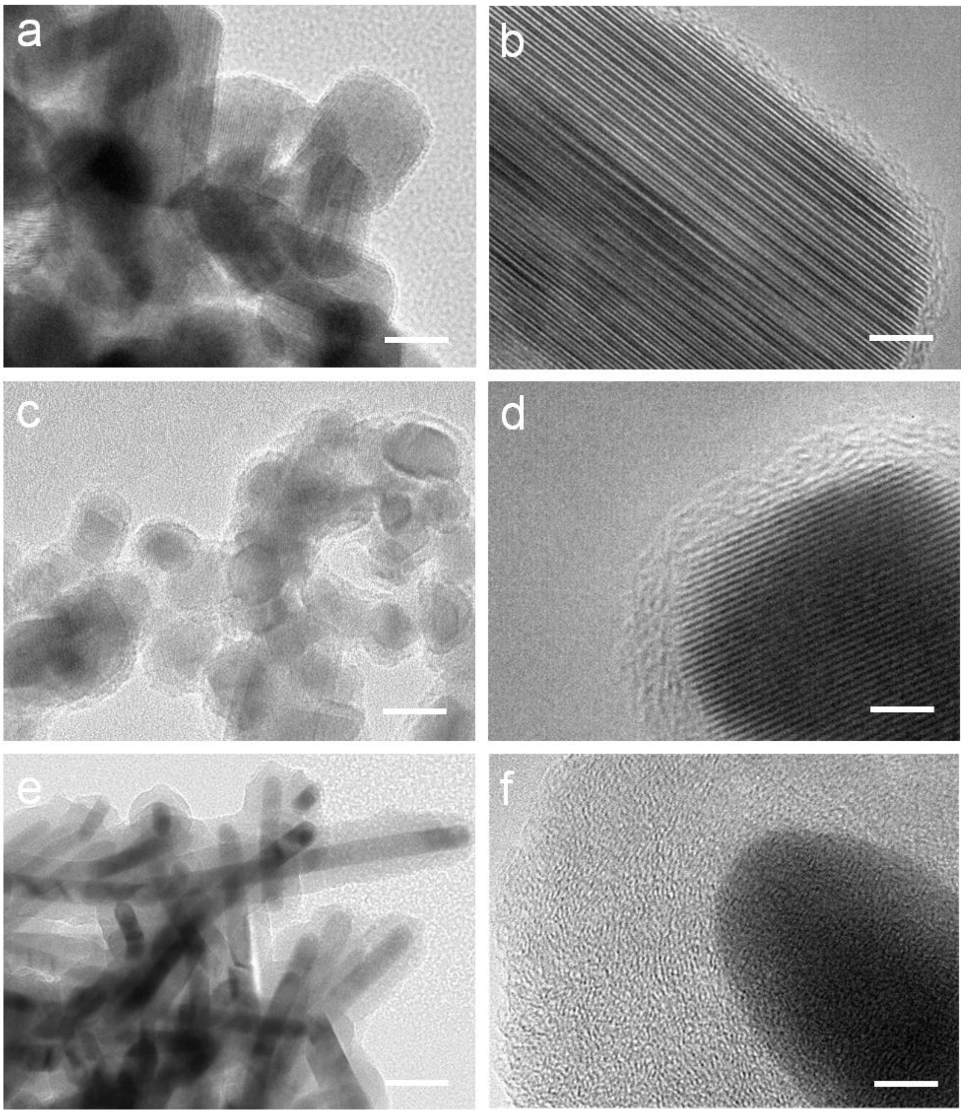

Supplementary Figure 8. Transmission electron microscopy analysis of other metal oxides. (a-b) TEM-HRTEM images of Nb_2O_{5-x}@C microspheres. (c-d) TEM-HRTEM images of TiO_{2-x}@C nanoparticles. (e-f) TEM-HRTEM images of ZnO_{-x}@C nanorods. Scale bars: (a, c) 20 nm, (e) 50 nm and (b, d, f) 5 nm.

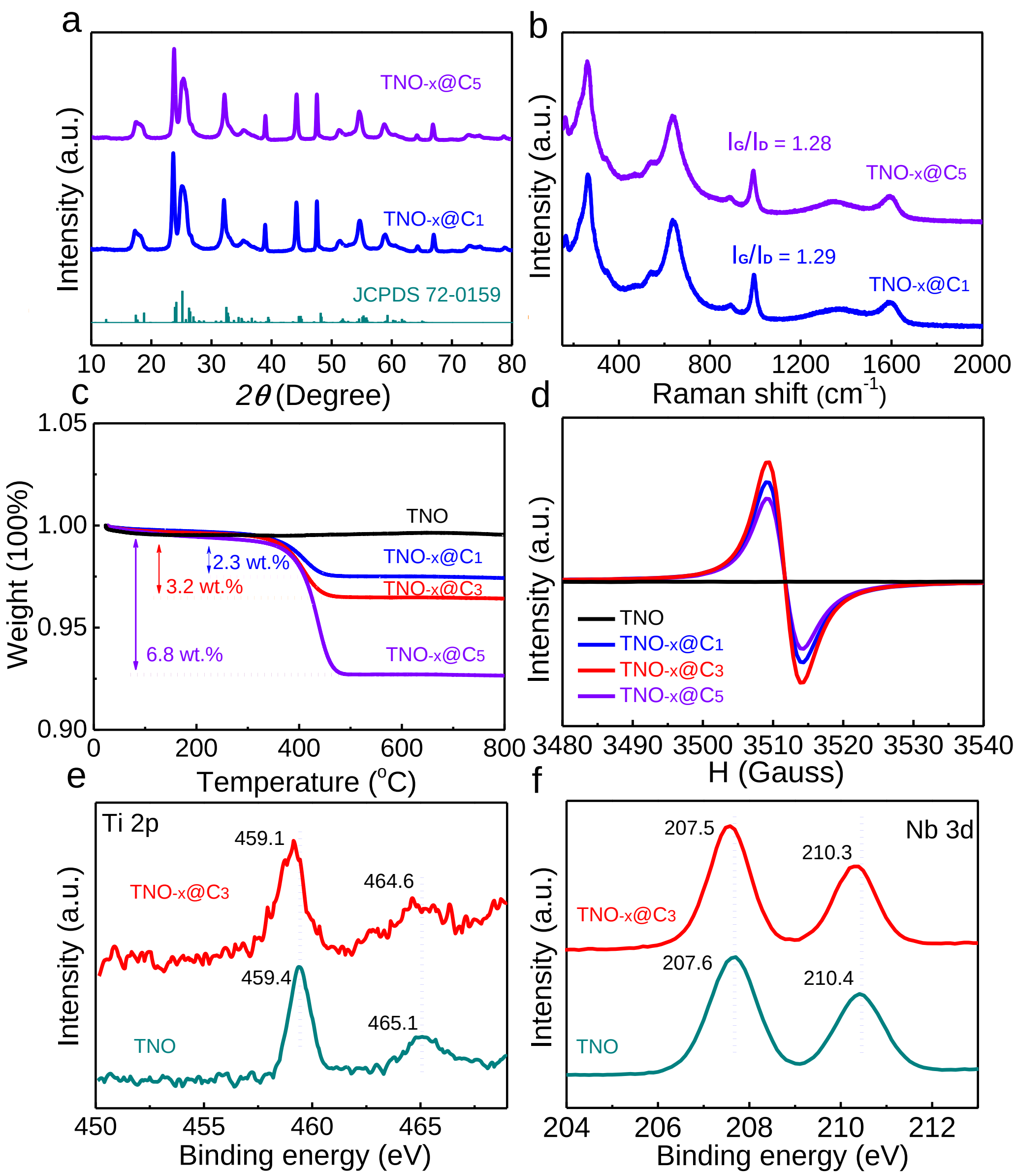

Supplementary Figure 9. Phase and composition characterization of these samples. (a) XRD pattern of $TNO_{-x}@C_1$ and $TNO_{-x}@C_5$ nanosheets. (b) Raman patterns of $TNO_{-x}@C_1$ and $TNO_{-x}@C_5$. (c) TG curves of four samples. (d) EPR spectra of four samples. (e) XPS spectra of Ti 2p in the pure TNO and $TNO_{-x}@C_3$. (f) XPS spectra of Nb 3d in the pure TNO and $TNO_{-x}@C_3$.

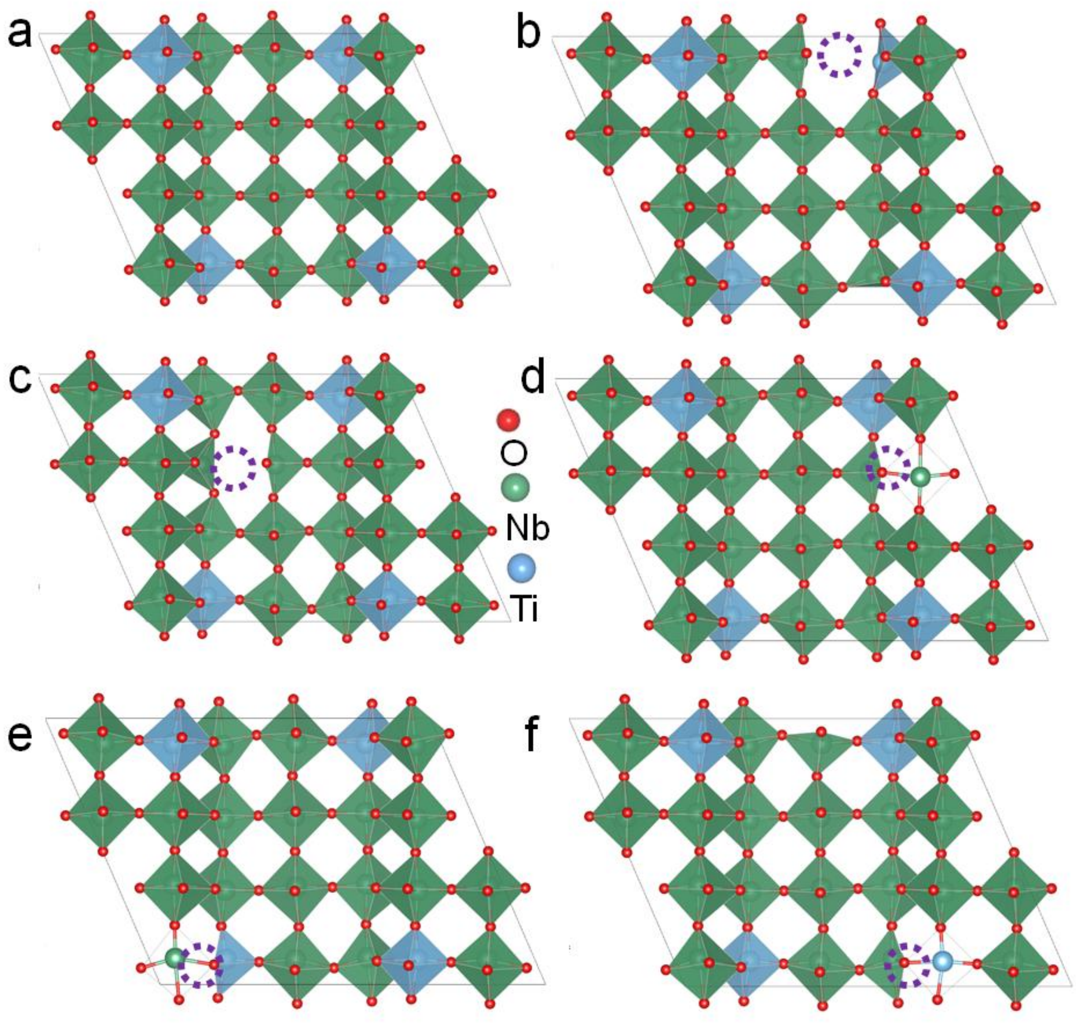

Supplementary Figure 10. The calculation models of TNO. (a) pure TNO, (b) defective TNO_{-x} (O:Ti-Nb), (c) defective TNO_{-x} (O:Nb-Nb), (d) defective TNO_{-x} (O:Nb-Nb-Nb), (e) defective TNO_{-x} (O:Nb-Ti-Nb) and (f) defective TNO_{-x} (O:Ti-Nb-Ti).

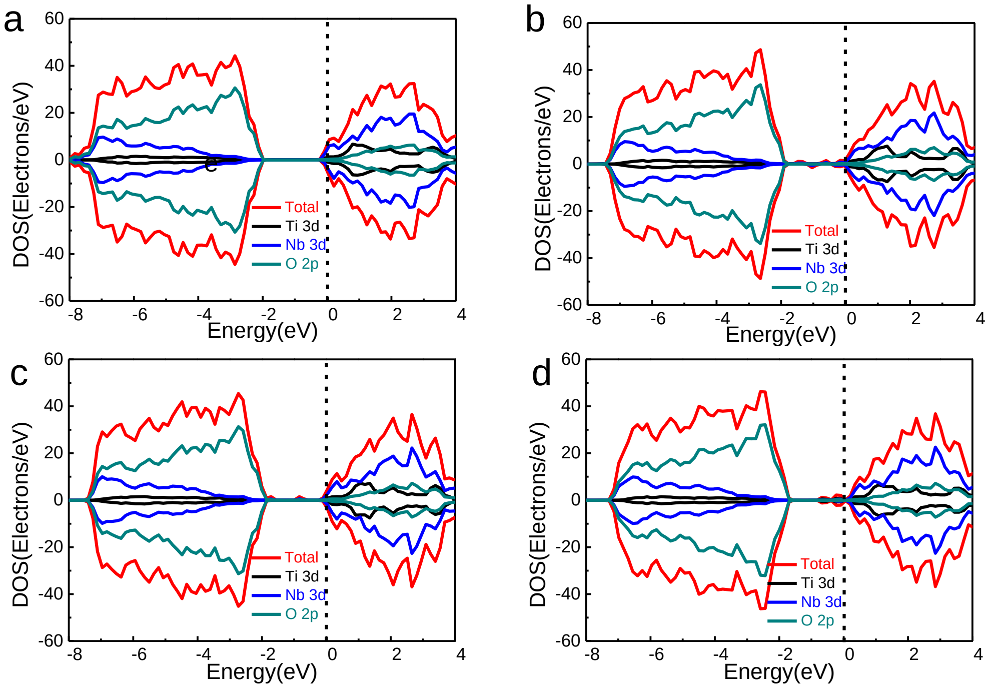

Supplementary Figure 11. Density of states (DOS). (a) defective TNO_{-x} (O:Nb-Nb), (b) defective TNO_{-x} (O:Nb-Nb-Nb)**,** (c) defective TNO_{-x} (O:Nb-Ti-Nb) and (d) defective TNO_{-x} (O:Ti-Nb-Ti).

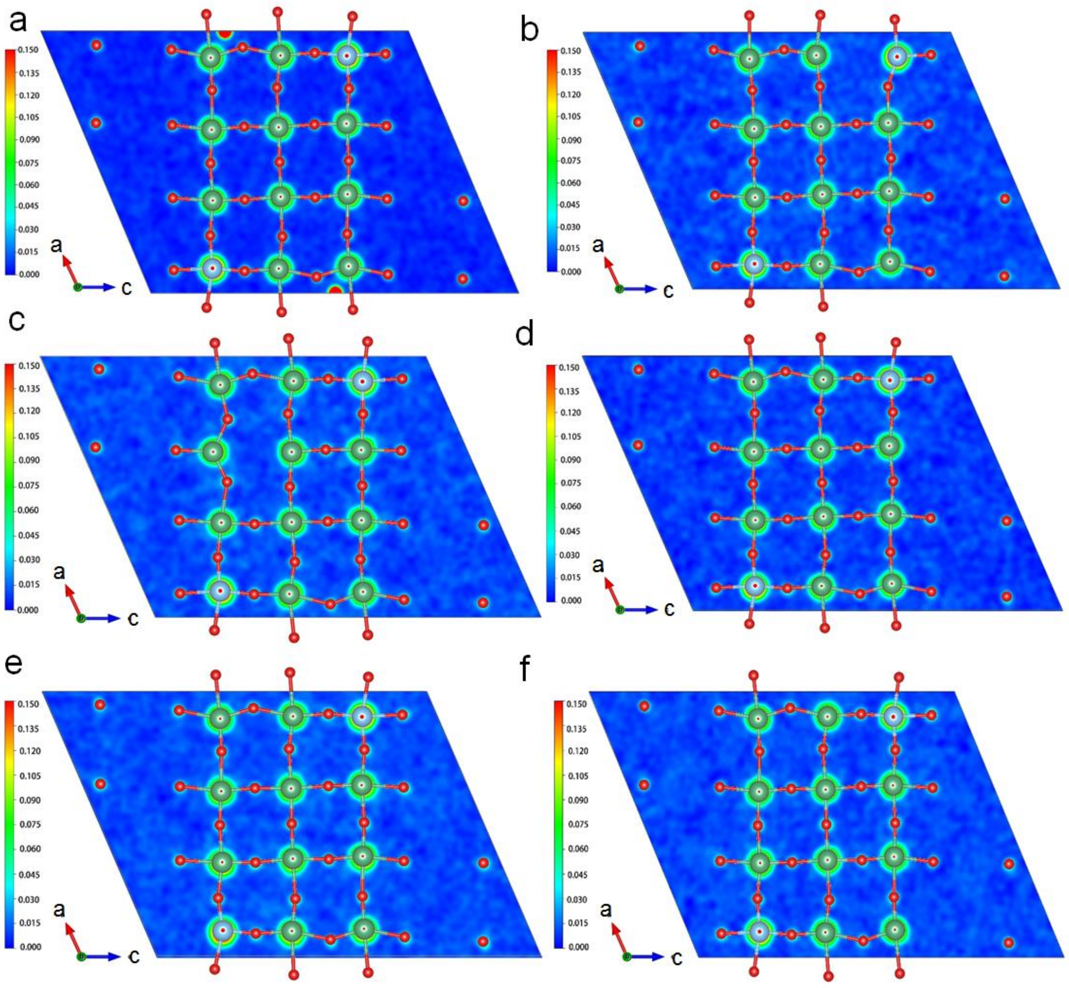

Supplementary Figure 12. The partial charge density plots in (010) plane. (a) pure TNO, (b) defective TNO_{-x} (O:Ti-Nb), (c) defective TNO_{-x} (O:Nb-Nb), (d) defective TNO_{-x} (O:Nb-Nb-Nb)**,** (e) defective TNO_{-x} (O:Nb-Ti-Nb) and (f) defective TNO_{-x} (O:Ti-Nb-Ti) (energy range from -0.5 eV to 0.5 eV).

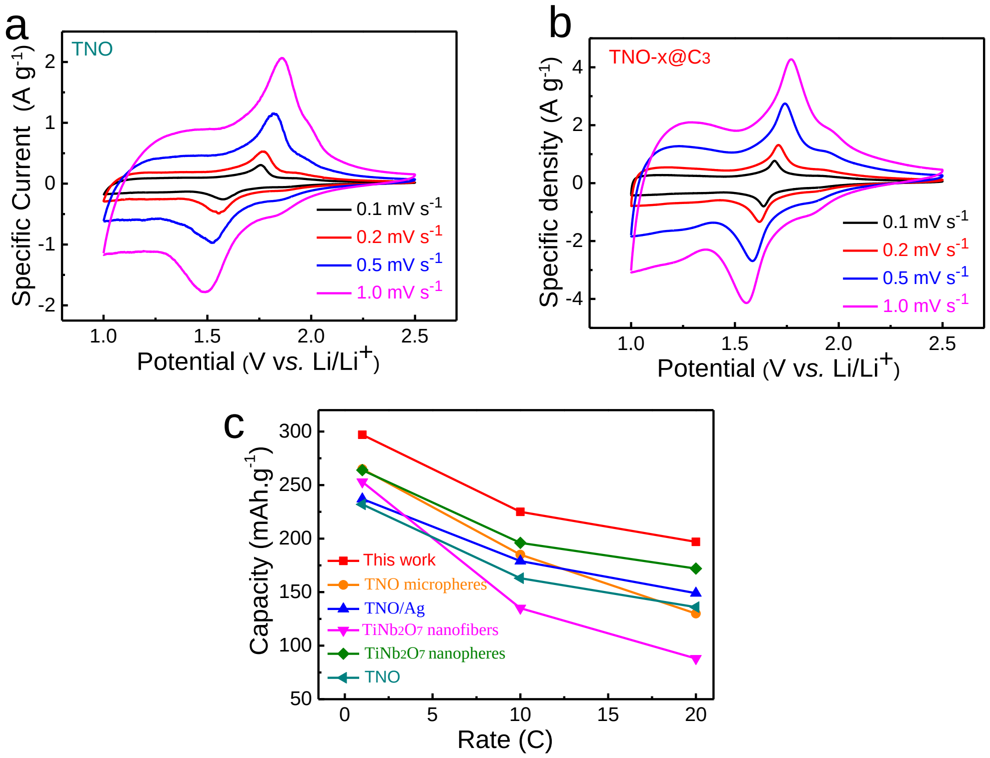

Supplementary Figure 13. Electrochemical characterization of TNO and TNO-$_x$@C$_3$ electrodes. (a-b) CV curves; (c) performance comparison.

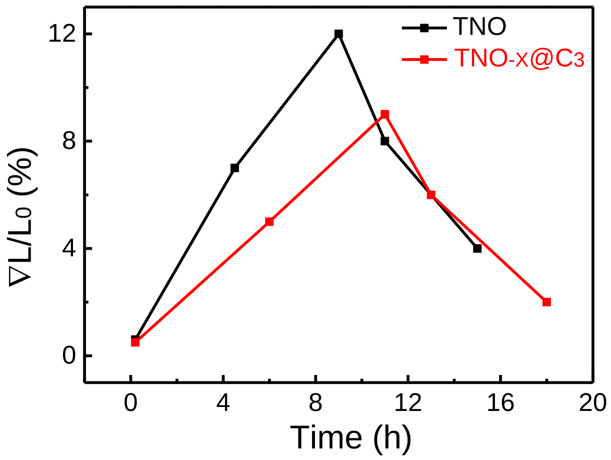

Supplementary Figure 14. Electrode thickness change analysis. Thickness change vs. time for TNO and TNO_{-x}@C_3 electrodes in a Galvanostatic test at 0.075 C.

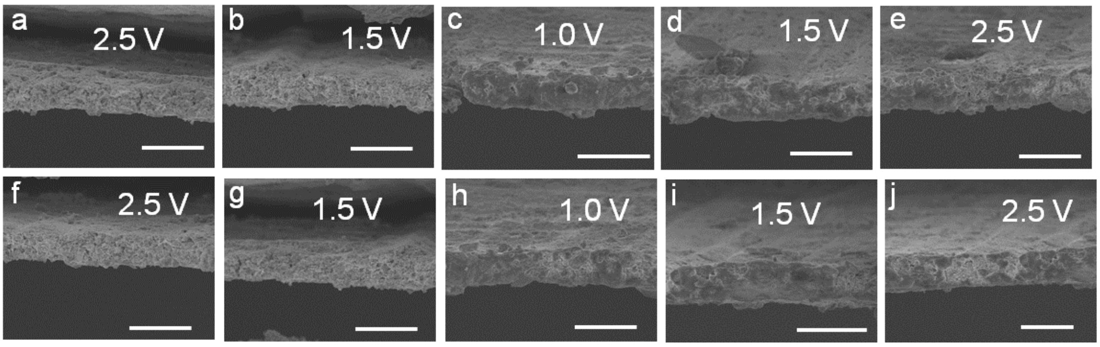

Supplementary Figure 15. Morphology characterization. SEM images of electrode thickness at the first cycle for (a-e) TNO and (f-j) TNO-x@C_3 electrodes at different potentials. Scale bars, 20 µm.

Supplementary Notes

Supplementary Note 1

The electrochemical performance of TNO and TNO$_{-x}$@C$_3$ electrodes was studied by cyclic voltammetry (CV) measurement at different scan rates in the voltage window of 1.0-2.5 V (**Figure S13**a-b). Note that both the TNO and TNO$_{-x}$@C$_3$ electrodes show large sharp redox peaks at 1.5-1.8 V (Nb^{4+}/Nb^{5+} redox couple) and small peaks at 1.8-2.0 V (Ti^{4+}/Ti^{3+} redox couple) as well as a broad bump peak at 1.0-1.5 V (Nb^{4+}/Nb^{3+} redox couple)[9,10]. Furthermore, the TNO$_{-x}$@C$_3$ electrode exhibits higher peak intensities and sharper CV peaks in comparison to pure TNO electrode, suggesting that TNO$_{-x}$@C$_3$ electrode demonstrates smaller polarization and higher electrochemical reactivity.

Supplementary Tables

Supplementary Table 1. The lattice parameters and energies of crystal structures.

Samples	a (Å)	b (Å)	c (Å)	α (º)	β (º)	γ (º)	Total energy
**TNO**	15.8288	3.8612	20.9686	90	113.3994	90	-733.6008
**TNO_{-x}(O:Ti-Nb)**	15.8337	3.8651	21.1053	90	113.4406	90	-724.3866
**TNO_{-x} (O:Nb-Nb)**	15.7006	3.8633	21.1322	90	113.7367	90	-724.5336
**TNO_{-x} (O:Nb-Nb-Nb)**	15.8134	3.8541	21.0007	90	113.4905	90	-723.8086
**TNO_{-x} (O: Nb-Ti-Nb)**	15.8130	3.8560	20.9889	90	113.3872	90	-723.9095
**TNO_{-x} (O:Ti-Nb-Ti)**	15.8333	3.8542	20.9871	90	113.4295	90	-723.6426

Supplementary Table 2. Results of crystal analysis of TNO and $TNO_{-x}@C_3$.

**Samples**	***a*** **(Å)**	**a_error**	***b*** **(Å)**	**b_error**	***c*** **(Å)**	**c_error**	***β*** **(º)**	***β*****_error**
TNO	15.5934	1.3E-4	3.7658	3E-5	20.1551	1.5E-4	113.7302	3E-4
$TNO_{-x}@C_3$	15.6103	1.4E-4	3.7666	3E-5	20.1635	1.7E-4	113.7473	5.3E-4

Supplementary Table 3. Comparison of electrochemical performance for samples

Active materials	Rate capability	Cyclability	Ref.
$TiNb_2O_7$ nanofibers	20 C (~ 150 mAh g^{-1})	50 cycles (1C, 90%)	1
Porous $TiNb_2O_7$	20 C (~190 mAh g^{-1})	1000 cycles (5C,87%)	2
Mo-doped $TiNb_2O_7$	10 C(~190 mAh g^{-1})	/	3
$Ti_2N_{10}O_{29}$ particles	20 C (120 mAh g^{-1})	800 cycles (10 C, 85%)	4
$Ti_2Nb_{10}O_{27.1}$	5 C (180 mAh g^{-1})	100 cycles (5C, 91.0%)	5
$Ti_2N_{10}O_{29}$	10C (130 mAh g^{-1})	/	6
$TiNb_2O_7$	2C (150 mAh g^{-1})	/	7
Porous $TiNb_{24}O_{62}$	20 C (181 mAh g^{-1})	500 cycles (10, 90.5%)	8
$Ru_{0.01}Ti_{0.99}Nb_2O_7$	5 C (181 mAh g^{-1})	100 cycles (5C, 90.5%)	9
$Ti_2N_{10}O_{29}$/C	10 C (194 mAh g^{-1})	100 cycles (5C, 89%)	10
$TiNb_6O_{17}$/C	10 C (199 mAh g^{-1})	500 cycles (10C, 82.9%)	11
TNO	20 C (135 mAh g^{-1})	500 cycles (10C, 85.9%)	Our work
TNO_{-x}@C_3	**20 C (197** mAh g^{-1}**)**	**500** cycles **(10C, 98.7%)**	**Our work**

Supplementary References

1. Tang, K., Mu, X. K., van Aken, P. A., Yu, Y. & Maier, J. "Nano-Pearl-String" $TiNb_2O_7$ as Anodes for Rechargeable Lithium Batteries. *Adv. Energy Mater.* **3**, 49-53 (2013).
2. Guo, B. K. *et al.* A long-life lithium-ion battery with a highly porous $TiNb_2O_7$ anode for large-scale electrical energy storage. *Energy Environ. Sci.* **7**, 2220-2226 (2014).
3. Song, H. & Kim, Y. T. A Mo-doped $TiNb_2O_7$ anode for lithium-ion batteries with high rate capability due to charge redistribution. *Chem. Commun.* **51**, 9849-9852 (2015).
4. Cheng, Q. S., Liang, J. W., Zhu, Y. C., Si, L. L., Guo, C. & Qian, Y. T. Bulk $Ti_2Nb_{10}O_{29}$ as long-life and high-power Li-ion battery anodes. J. Mater. Chem.A **2**, 17258-17262 (2014).
5. Lin, C. F. *et al.* Defective $Ti_2Nb_{10}O_{27.1}$: an advanced anode material for lithium-ion batteries. *Sci. Rep.* **5**, 17836-17836 (2015).
6. Wu, X. Y. *et al.* Investigation on $Ti_2Nb_{10}O_{29}$ anode material for lithium-ion batteries. *Electrochem. Commun.* **25**, 39-42 (2012).
7. Han, J. T., Huang, Y. H. & Goodenough, J. B. New anode framework for rechargeable lithium batteries. *Chem. Mater.* **23**, 2027-2029 (2011).
8. Yang, C. *et al.* Porous $TiNb_{24}O_{62}$ microspheres as high-performance anode materials for lithium-ion batteries of electric vehicles. *Nanoscale* **8**, 18792-18799 (2016).

9. Lin, C. F. *et al.* $Ru_{0.01}Ti_{0.99}Nb_2O_7$ as an intercalation-type anode material with a large capacity and high rate performance for lithium-ion batteries. *J. Mater. Chem.A* **3**, 8627-8635 (2015).

10. Liu, G. Y. *et al.* Synthesis of $Ti_2Nb_{10}O_{29}$/C composite as an anode material for lithium-ion batteries Int. J. Hydrogen Energy **41**, 14807-14812 (2016).

11. Mao, W. T. *et al.* Synthesis of $TiNb_6O_{17}$/C composite with enhanced rate capability for lithium ion batteries. *Ceram. Int.* **42**, 16935-16940 (2016).
